# Investigation on the role of the molecular weight of polyvinyl pyrrolidone in the shape control of high-yield silver nanospheres and nanowires

**DOI:** 10.1186/1556-276X-9-17

**Published:** 2014-01-13

**Authors:** Yuan-Jun Song, Mingliang Wang, Xiao-Yang Zhang, Jing-Yuan Wu, Tong Zhang

**Affiliations:** 1 School of Chemistry and Chemical Engineering, Southeast University, Nanjing 211189, People’s Republic of China; 2 School of Electronic Science and Engineering, Key Laboratory of Micro-Inertial Instrument and Advanced Navigation Technology, Ministry of Education, Southeast University, Nanjing 210096, People’s Republic of China; 3 Suzhou Key Laboratory of Metal Nano-Optoelectronic Technology, Suzhou Research Institute of Southeast University, Suzhou 215123, People’s Republic of China

**Keywords:** Polyvinyl pyrrolidone, Molecular weight, Nanowire, Nanosphere

## Abstract

Serving as shape control agent, polyvinyl pyrrolidone (PVP) has been widely used in chemical synthesis of metal nanoparticles. However, the role of molecular weight (MW) of PVP has been rarely concerned. In this study, we show a facile method to control the shapes of silver nanocrystals using PVP with different MWs. PVP_MW=8,000_, PVP_MW=29,000_, PVP_MW=40,000_, and PVP_MW=1,300,000_ are compared in the present study. Surprisingly, high-yield silver rodlike nanostructures, nanospheres, and nanowires can be obtained under the same growth environment and reactant concentrations by simply changing the MW of PVP. The mechanism studies of the role of PVP with different MWs in the growth process were carried out systemically using the morphology and spectroscopic measurement, FT-IR spectrum analysis, and seed crystallization monitoring. The results indicate that the MW of PVP plays a determinant role in the morphology and optical property control of the silver nanocrystals. Meantime, the concentration of PVP was found to be an assistant factor to further improve the shape and the yield of the synthesized nanocrystals.

## Background

The synthesis of metal nanoparticles with high uniformity attracts considerable attentions due to their fantastic optical properties arising from localized surface plasmon resonance (LSPR) [1-3]. Such plasmonic nanoparticles, especially silver, are widely used in catalysis [4,5], biological and chemical sensors [6-8], and surface-enhanced Raman spectroscopy [9-11]. It has been recognized that the optical spectral signatures of plasmonic nanoparticles are primarily dependent on their shapes [12-14]. Leading works in the synthesis of silver nanoparticles have focused on the shape control of silver nanocrystals via various routes. Wiley et al. [15] controlled the shapes of silver nanocrystals by varying reaction conditions such as the precursor concentration, molar ratio of the surfactant, and silver ions. As well known, the final structure of the nanocrystals are mainly determined by the crystallinity of seeds produced in the early stage of the reaction. Xia's group prepared silver pentagonal nanowires, nanocubes, and bipyramids from multiply twinned decahedral seeds, single-crystalline seeds, and single-twinned seeds, respectively [16]. As for the crystals' control of seeds, Xia et al. introduced Cl^-^ or Br^-^ as etchants combined with oxygen to avoid the formation of undesired seeds [17]. Another factor that influences the shape uniformity of the nanocrystals is self-nucleation in the reaction process. Self-nucleation of reductive silver atoms usually blocks the seed growth process resulting in the formation of spherical by-productions. The solution to the problem is to decrease the reduction rate of silver ions. Zhang et al. [18] applied a weak reductant to control the reduction rate. Meantime, citrate ligands used can also decrease the reduction rate because of complexation between silver ions and citrate ligands. Using polyol reduction method in the presence of polyvinyl pyrrolidone (PVP), Sun and co-workers successfully prepared silver nanowires [19-22]. Alternatively, the addition of as-prepared seeds [19] in the initial growth step has been suggested to induce the formation of nanowires preferentially. However, these reaction processes are usually complex or difficult to control. Without fine control of reactant concentrations and growth process, the obtained silver nanowires are always in low yield accompanied by large amounts of by-products such as nanocubes or nanospheres growing from isotropic seeds. In these cases, the post processing, such as low rotation-rate centrifugation [20] or special separation technique [23] to purify nanowires, is usually indispensable. Therefore, it is highly desirable to develop a reliable and facile method for the synthesis of silver nanocrystals in high yield with uniform size.

In the polyol process, acting as stabilizer, PVP plays an important role in controlling the shape. Chou et al. [24] compared the ability of PVP to stabilize silver colloids in the presence of NaOH or Na_2_CO_3_. Liu et al. [25] also proposed that the crystal structure shape was related to the capping modes between PVP with different molecular weights (MWs) and silver nanocrystals. Although the changes arising from the addition of PVP with different MWs have been observed in previous works, the exact function of the MW of PVP on the formation of silver nanocrystals has not been clarified until now. In this work, we deeply studied the role of MW of PVP in the shape control of silver nanocrystals. According to optical spectroscopic analysis and statistic of the yield and average size of each product prepared by varying the MW and concentration of PVP, we obtained the relationship between the MW of PVP and preferential products. By analyzing the interaction between PVP with different MW and silver crystals by Fourier transform infrared (FT-IR)spectroscopy, we deduce the role of PVP in the nucleation and growth processes. The results suggest that we provide a facile and robust strategy for the synthesis of well-shaped silver nanocrystals in high yield.

## Methods

Silver nitrate (AgNO_3_ 99 + %), sodium chloride (NaCl), and ethylene glycol (EG) were all purchased from Nanjing Chemical Reagent Co. Ltd (Nanjing, People's Republic of China). Polyvinyl pyrrolidone (PVP_MW=8,000_, PVP_MW=1,300,000_) were purchased from Aladdin (Shanghai, People's Republic of China). PVP_MW=29,000_ and PVP_MW=40,000_ were purchased from Sigma-Aldrich (St. Louis, MO, USA).

We used a colloidal synthesis method improved from the literature [26]. The method is one of the main methods for silver nanowire preparation. However, we found that when PVP_MW=40,000_ was used in this method, there are always plenty of by-products such as nanospheres and nanocubes unless the reaction condition was strictly controlled. It provides us an opportunity to exhibit the role of MW and the concentration of PVP in the synthesis process using this method. In each synthesis, l-mL EG solution of AgNO_3_ (0.9 M) and 0.6-mL EG solution of NaCl (0.01 M) were added into 18.4-mL EG solution of PVP (0.286 M). Then, the mixture was refluxed at 185°C for 20 min. After these processes, the excess PVP and EG were removed by adding deionized water centrifuged at 14,000 rpm for 10 min, three times. The centrifugation ensures that all the products can be collected for the sake of statistics of shapes and size.

The morphologies of the prepared silver samples were observed by transmission electron microscopy (TEM; JEM-2100, JEOL Ltd., Akishima, Tokyo, Japan) and scanning electron microscopy (SEM; SIRION, Durham, NH, USA). FT-IR analysis was conducted on the FT-IR spectrum (NICOLET 5700, Thermo Fisher Scientific, Waltham, MA, USA). UV-visible near-infrared (NOR) spectra were recorded by a fiber-optic spectrometer (PG2000, Ideaoptics Technology Ltd., Shanghai, People's Republic of China).

## Results and discussion

### Morphology characterization

**Figure 1:**
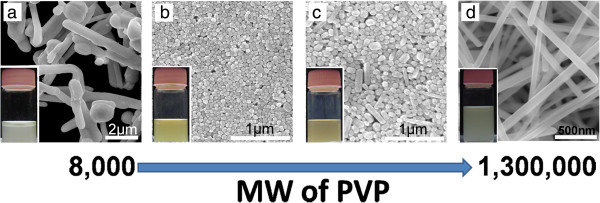
**SEM images of silver nanocrystals synthesized using PVP with varying MWs.** Varying MWs **(a)** 8,000, **(b)** 29,000, **(c)** 40,000, and **(d)** 1,300,000. The insets are photographs of the corresponding silver colloids.

The experimental results shown in Figure 1 indicate that the MW of PVP plays a key role in the shape control of silver nanocrystals. Figure 1 shows a series of silver nanocrystals prepared in the presence of PVP with different MWs. The inset pictures were taken in a dark room under the exposure of white LED panel light from the bottom which is similar to natural light having a wide spectral range. Different colors of silver colloids corresponding to different morphologies can be observed easily. Figure 1a presents the rodlike silver nanostructures synthesized using PVP_MW=8,000_. As shown in Figure 1a, two or more silver nanorods are melded together randomly in several types such as end-to-end, end-to-side, or parallel nanojoint, which has potential applications in nanocircuits [27]. Such typical morphology corresponds to the white color colloids that can be seen from the photograph in the inset of Figure 1a. When PVP_MW=29,000_ was used, a generation of bright yellow-green colloids was observed as shown in the inset of Figure 1b. The SEM image indicates that such color corresponds to the formation of high-yield silver nanospheres with uniform size around 60 nm [28]. Apparently, it provides a facile method for the synthesis of monodisperse silver nanospheres with high uniformity using PVP_MW=29,000_. Colloids in the inset of Figure 1c appear to be a muddy and dark yellow color when PVP_MW=40,000_ was used which is similar to that of the inset in Figure 1b. The reason is that the two colloids both have absorption of blue light shown in extinction spectra which will be discussed in the next Section. A large number of nanoparticles and a small amount of nanowires are observed in Figure 1c. However, the morphologies of silver nanoparticles are irregular and the sizes are nonuniform. It indicates that monodisperse silver particles with uniform shape and size can be hardly obtained when PVP_MW=40,000_ was used as a capping agent in the current synthesis process. When PVP_MW=1,300,000_ was used, it can be seen clearly that high-yield (>90 %) silver nanowires were obtained, as shown in Figure 1d. The color of silver colloids is yellowish white, similar to the highly purified silver nanowire colloids obtained after cross-flow filtration [23]. Although the color in the inset of Figure 1d is similar to that of in Figure 1a, the extinction spectrum of rodlike nanocrystal has a broad scattering band from the visible to the near-infrared wavelengths, while the extinction spectrum of silver nanowire has absorption of short wavelength light leading to exhibiting yellowish white rather than white. Compared with the result of Tsuji et al. [26], we can synthesize silver nanowires in higher yield using a simpler and faster method which obviates bubbling O_2_ and controlling the heating up time from room temperature to 185°C.

**Figure 2:**
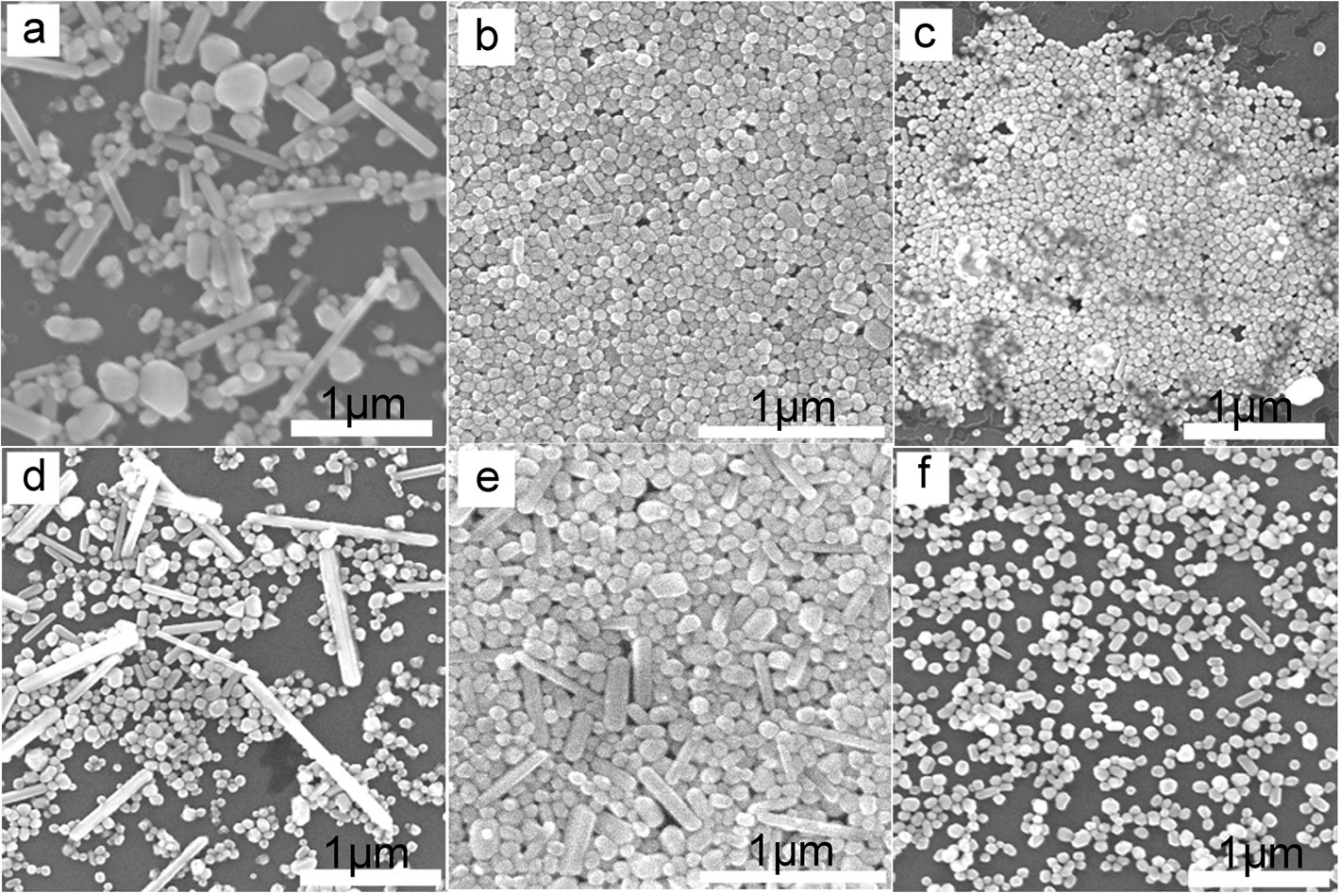
**SEM images of silver nanocrystals obtained by varying the concentrations of PVP**_**MW=29,000 **_**and PVP**_**MW=40,000**_**.** PVP_MW=29,000_**(a)** 0.143 M, **(b)** 0.286 M, and **(c)** 0.572 M. PVP_MW=40,000_**(d)** 0.143 M, **(e)** 0.286 M, and **(f)** 0.572 M.

The concentration dependence of PVP in the synthesis is also investigated. Table 1 presents the yield and average size of each product prepared by varying the concentrations of PVP with MWs of 29,000, 40,000, and 1,300,000. Figure 2 shows the SEM images of silver nanoparticles prepared at different concentrations of PVP_MW=29,000_. It can be observed that in Figure 2a, 15% silver nanowires and other various shapes of nanoparticles were obtained at a concentration of 0.143 M. When the concentration of PVP was 0.286 M, high-yield nanospheres with about 1% nanowires were prepared as shown in Figure 2b. Figure 2c shows that the average size of nanospheres was smaller with 0.572 M PVP due to the high concentration offering a stronger stable ability to prevent the aggregation of nanoparticles. The same trend can be seen in Figure 2d,e which shows the SEM images of silver nanoparticles obtained using PVP_MW=40,000_ with different concentrations of PVP. We found that the yield of silver nanowires was about 20%, 5%, and 1% at concentrations of 0.143, 0.286, and 0.572 M, respectively. Figure 2 indicates that with the increase of concentration of PVP, the shape and size of silver nanoparticles became more uniform. The reason may be that a higher concentration of PVP forms a thicker coating over the surface of silver nanoparticles leading to a weaker selective adsorption of PVP which induces isotropic growth into the nanospheres [29].

**Table 1:** Statistic of the yield and average size of each product prepared by varying concentrations of PVP

**Concentration of PVP (M)**		**Nanowire**		**Nanospheres**
		**Yield (%)**	**Diameter (nm)/length (μm)**	**Diameter (nm)**
PVP_MW=29,000_	0.143	15	100 ± 10/1 ± 0.5	100 ± 20
	0.286	1	100 ± 10/0.6 ± 0.1	60 ± 10
	0.572	1	100 ± 10/0.4 ± 0.1	50 ± 10
	0.143	20	100 ± 10/1.5 ± 0.2	100 ± 50
PVP_MW=40,000_	0.286	5	100 ± 10/0.6 ± 0.1	100 ± 50
	0.572	1	100 ± 10/0.6 ± 0.1	60 ± 10
	0.143	90	200 ± 100/2 ± 0.5	200 ± 50
PVP_MW=1,300,000_	0.286	95	100 ± 20/4 ± 2	200 ± 50
	0.572	95	100 ± 10/6 ± 1	200 ± 50

With MW of 29,000; 40,000; and 1,300,000.

**Figure 3:**
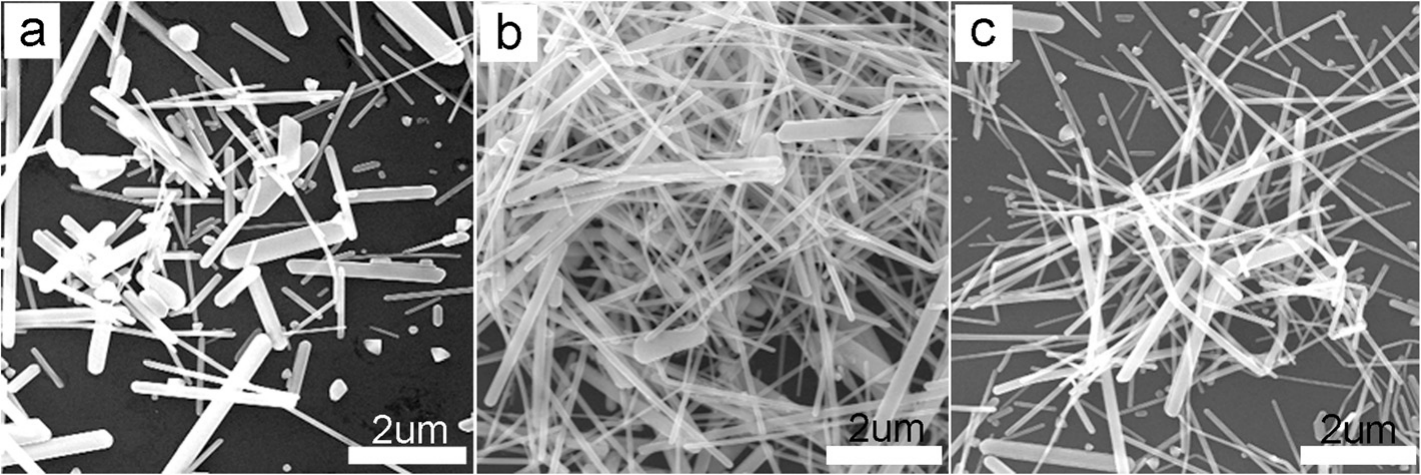
**SEM images of silver nanocrystals obtained by varying the concentration of PVP**_**MW=1,300,000**_**. (a)** 0.143 M, **(b)** 0.286 M, and **(c)** 0.572 M.

Figure 3 presents SEM images of silver nanocrystals obtained at different concentrations of PVP using PVP_MW=1,300,000_. By comparing three SEM images of Figure 3, one can see that the concentration of PVP has less influence on the yield of silver nanowires when PVP_MW=1,300,000_ was used. However, it is found that the concentration of PVP contributes to the control of diameter of the synthesized nanowire. In Figure 3a, there are short nanorods, long nanowires, and some nanoparticles (<10%). Figure 3b shows the yield of silver nanowires with uniform diameter and length increased to about 95% which is similar to the result shown in Figure 3c. From the above comparison study, it should be noted that varying the MWs of PVP is more efficient on the shape control of silver nanocrystals than varying the concentrations of PVP.

### Optical property characterization

**Figure 4:**
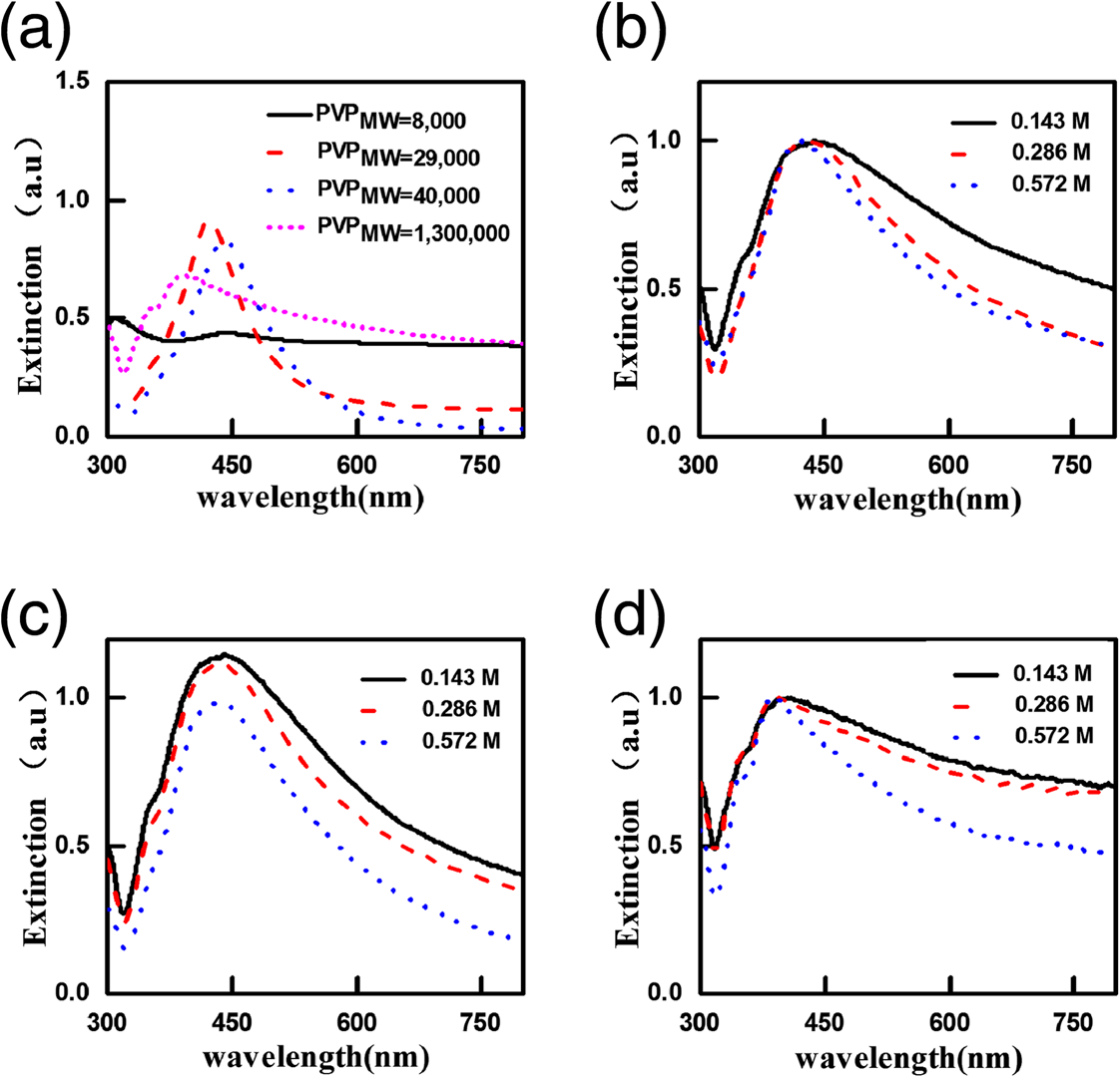
**The optical characteristics of the silver solution. (a)** The extinction spectra of the silver nanostructure solution obtained with different PVPs of 0.286 M. **(b)** The extinction spectra of the silver nanostructure solution obtained at different concentrations of PVP_MW=29,000_, **(c)** The extinction spectra of the silver nanostructure solution obtained at different concentrations of PVP_MW=40,000_. **(d)** The extinction spectra of the silver nanostructure solution obtained at different concentrations of PVP_MW=1,300,000_.

UV-visible NIR spectrophotometer can also be used to confirm the morphologies of silver nanocrystals. The resonance bands of the plasmonic nanocrystals are mainly dependent on the distribution of the electromagnetic field on the surface of the metal nanocrystals. In other words, metal nanoparticles with different shapes and sizes should have different optical signatures. Figure 4a exhibits the extinction spectra of the silver solution with different PVPs at 0.286 M. As shown in Figure 4a, the rodlike shape prepared with PVP_MW=8,000_ has a broad scattering band from the visible to the near-infrared wavelengths leading to the white color shown in the inset in Figure 1a. Because the structure joined together can trap light effectively [30], such rodlike nanostructure can be used as a hot spot. The extinction spectra of the silver nanostructure solution using PVP_MW=29,000_ have a main resonance peak at 430 nm and a shoulder peak at 360 nm corresponding to the nanosphere [17]. In comparison, that of PVP_MW=40,000_ exhibits a redshift and broader absorption range ascribed to the irregular shapes of the products. In the extinction spectrum of the solution with PVP_MW=1,300,000_, there are two resonance peaks at 390 and 350 nm belonging to the optical signature of silver nanowire [19].

Figure 4b,c,d shows the extinction spectra of the silver nanostructure solution obtained at different concentrations of PVP_MW=29,000_, PVP_MW=40,000_, and PVP_MW=1,300,000_, respectively. We find that when the concentration of PVP increases, the resonance peaks blue-shifted and extinction bands became narrow. The reason is that with the decrease of the nanoparticle size, the resonance peak will shift towards the shorter wavelength and uniform size will cause narrow extinction bands [31], which correspond to our experimental results.

### Supporting evidence for the function of MW of PVP

**Figure 5:**
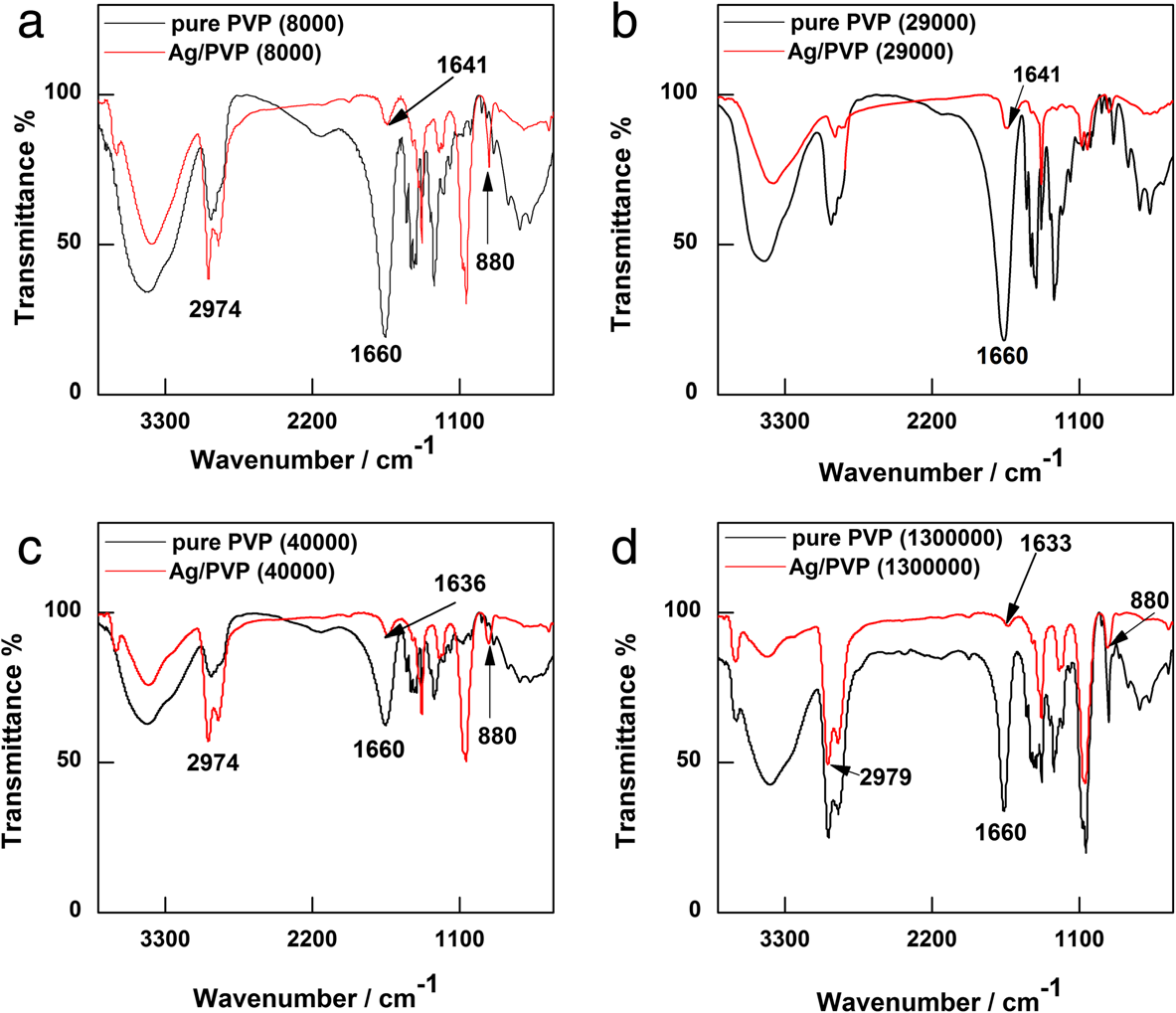
**FT-IR spectra of pure PVP and Ag/PVP with different MWs. (a)** MW = 8,000. **(b)** MW = 29,000. **(c)** MW = 40,000. **(d)** MW = 1,300,000.

In this section, we show the reason why PVP can affect the silver nanostructure, and it is because PVP prefers to adsorb on the (100) facets of silver nanocrystals in EG [32]. The interaction process can be given by Equation 1. To determine the strength of adsorption between Ag^+^ ions and different PVPs, we resort to FT-IR analysis. Figure 5 presents the FT-IR spectra of pure PVP and Ag/PVP. In the spectra of pure PVP, the absorption peak locates at around 1,660 cm^-1^ ascribed to the stretching vibration of C = O which is slightly dependent on the MW of PVP. Compared with the free C = O stretching band of pure PVP, the adsorption peaks of Ag/PVP all shift towards the lower wave number due to the coordination between Ag^+^ ions and carbonyl oxygen. The positions of free and coordinated C = O bands in Ag/PVP with four kinds of MW are shown in Table 2. Because the strength of the coordination interaction between Ag^+^ ions and PVP can be estimated in terms of the magnitude of band shifts [33], the sequence of the strength of the coordination interaction between Ag^+^ ions and PVP occurs as follows: PVP_MW=1,300,000_ > PVP_MW=40,000_ > PVP_MW=8,000_ > PVP_MW=29,000_. The larger extent of blue shift band indicates a stronger selective adsorption on the (100) facets of silver nanocrystals, which is one of the important factors giving rise to the different morphologies of silver nanocrystals produced with different PVPs. As can be seen in Figure 5a,c,d, there is a peak at about 880 cm^-1^ assigned to the breathing vibration of the pyrrolidone ring, indicating that the pyrrolidone ring may be tilted on the surface of silver nanowires [34]. In addition, in these three figures, the peak at 2,970 cm^-1^ ascribed to asymmetric stretching vibration of CH_2_ in the skeletal chain of PVP, which implies that the CH_2_ chain is close to the surface of silver nanowires. Therefore, the conformation of PVP makes the fine and close adsorption on the (100) facets of silver nanocrystals. Conversely, both peaks in Figure 5b are weak, leading to the formation of high-yield silver nanospheres which is consistent with the result shown in Figure 1b.(1)

**Table 2:** Positions of free and coordinated C = O bands in Ag/PVP with four kinds of MWs

**System**	**MW**			
	**8,000**	**29,000**	**40,000**	**1,300,000**
FT-IR (cm^-1^)	1,640	1,644	1,636	1,633
Redshift (cm^-1^)	20	16	24	27

Another factor influencing the morphology of silver nanocrystals with different PVPs is the steric effect. Shorter chains of PVP cause a smaller steric effect which can combine PVP with silver nanoparticles in the colloid better but also results in incomplete coating of silver nanocrystals. In this case, silver nanocrystals may aggregate together. On the contrary, PVP with longer chains can protect silver nanocrystals from aggregation. However, a thicker coating on the surface of silver nanocrystals may decrease the strength of the coordination interaction between Ag^+^ ions and PVP.

Thus, considering the combined effect of chemical adsorption and steric effect, we can deduce the growth mechanism of silver nanocrystals with these four PVPs. The formation process of silver nanocrystals can be divided into three stages. In the first stage, Ag^+^ ions were reduced by EG following the reaction in Equations 2 and 3. Then, silver nucleus formed with the protection of PVP. As soon as the color of the solution changed, the seeds began to exit. The last step is the growth of silver nanocrystals with the protection of PVP:

(2) $$2\mathrm{HOC}H_{2}CH_{2}\mathrm{OH}\to 2CH_{3}\mathrm{CHO}+2H_{2}0$$

(3) $$\begin{array}{c} 2Ag^{+}+CH_{3}\mathrm{CHO}+H_{2}O\to CH_{3}\mathrm{COOH}+2\mathrm{Ag}\\ \;+2H^{+} \end{array}$$

**Figure 6:**
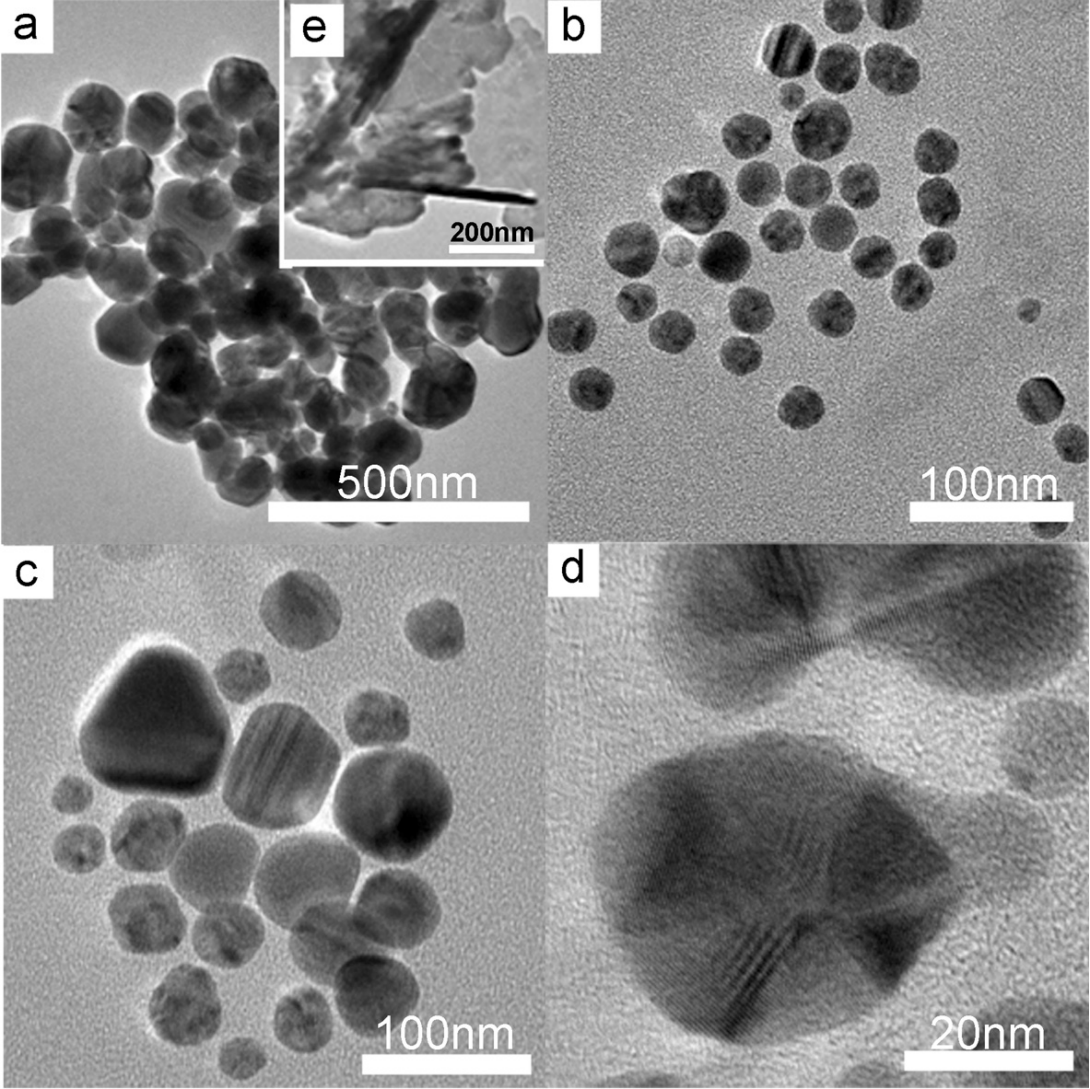
**TEM images of silver nanocrystals prepared in the presence of PVP with different MWs at 100°C. (a)** MW = 8,000. **(b)** MW = 29,000. **(c)** MW = 40,000. **(d)** MW = 1,300,000. **(e)** TEM image of silver nanostructure prepared at 110°C using PVP_MW=8,000_.

It is well known that the morphologies of silver nanocrystals strongly depend on the seeds formed in the initial stage. In order to compare the seeds in the presence of different PVPs visually, we prepared seeds at 100°C at the PVP of 0.286 M without any change of other conditions. Figure 6 shows the silver nanoparticles prepared at 100°C with different PVPs. The shortest PVP_MW=8,000_ are easier to cover with the surface of silver nucleus than other PVPs because of the smallest steric effect resulting in a stronger adsorption interaction between the PVP and silver nucleus. However, PVP_MW=8,000_ has less power to go against the aggregation of nanoparticles; thus, in Figure 6a, these silver nanoparticles gathered together. With the increased temperature, some of the nanoparticles grew into nanowires while others aggregated into plates which can be observed in Figure 6e. Because the activity of the end of nanowires without coverage of PVP is high [35], it would be likely to form an end-to-end or end-to-side connection of silver nanowires, except that some silver nanowires may aggregate in a parallel way.

Compared with PVP_MW=8,000_, PVP_MW=29,000_ with longer chains is able to offer more protection against aggregation, but weakest selective adsorption of PVP on the (100) facets of silver nanocrystals leads to the formation of isotropic seeds. Hence, in Figure 6b, one can see seeds prepared at 100°C mainly involving quasi-spherical seeds. Finally, these seeds evolved into nanospheres. The moderate selective adsorption of PVP_MW=40,000_ on the (100) facets results in exits of anisotropic seeds such as nanoplate and twinned pentahedron as shown in Figure 6c. Because each facet has different growth resistances, in different conditions, silver seeds evolve into different shapes [16]. According to our observations, this is the main reason why PVP_MW=40,000_ is the commonest capping agent used for the preparation of silver nanoparticles. However, any undesired disturbance can greatly influence the morphologies of silver nanocrystals. For example, Tsuji et al. [26] demonstrated that there was a significant difference in the yield and average size of silver nanowires when they varied the reaction temperature or reaction atmosphere with PVP_MW=40,000_. As a result, although numerous nanocrystals have been obtained, PVP_MW=40,000_ is not the best choice for high-yield synthesis of silver nanocrystals due to limitations in production efficiency, yield, and reproducibility. PVP_MW=1,300,000_ has both the strongest interaction of PVP on the surface of silver nanocrystals and the ability of anti-agglomeration arising from longest chains, inducing the formation of twinned pentahedron seeds which can be observed in Figure 6d. According to the growth mechanism of silver nanowires reported by Xia et al. [29], twinned pentahedron seeds will evolve into nanowires finally.

## Conclusions

In this study, we exhibit that the MW of PVP plays a critical role in the shape control of silver nanocrystals. The function of PVP on the shape control of silver nanocrystals can be discussed from two aspects: adsorption effect and steric effect. Results suggest that adsorption effect holds the dominated position in the selective adsorption of PVP on (100) facets of silver nanocrystals when the MW of PVP is very small, while with the increase of MW, the chemical adsorption gradually takes the place of the former. Therefore, different silver nanocrystals can be obtained by varying MWs of PVP. In addition, compared with the products obtained by varying the concentrations of PVP, we find that the MW of PVP plays a more efficient role in shape control. Our study on the effect of PVP with different MWs paves the way for the synthesis of silver monodisperse nanospheres and nanowires in high yield.

## Competing interests

The authors declare that they have no competing interests.

## Authors’ contributions

YJS carried out the main part of synthetic and analytic works and drafted the manuscript. XYZ and JYW participated in synthetic and analytic works. MLW and TZ participated in the discussion of experimental details and participated in the draft preparation. All authors read and approved the final manuscript.
